# A comparison of imputation procedures and statistical tests for the analysis of two-dimensional electrophoresis data

**DOI:** 10.1186/1477-5956-8-66

**Published:** 2010-12-15

**Authors:** Jeffrey C Miecznikowski, Senthilkumar Damodaran, Kimberly F Sellers, Richard A Rabin

**Affiliations:** 1 Department of Biostatistics; University at Buffalo, Buffalo, NY 14214 USA; 2 Department of Pharmacology and Toxicology; School of Medicine and Biomedical Sciences, University at Buffalo, Buffalo, NY 14214 USA; 3 Department of Mathematics and Statistics; Georgetown University, Washington, DC 20057 USA; 4 Department of Biostatistics; Roswell Park Cancer Institute, Buffalo, NY 14263 USA

## Abstract

**Background:**

Numerous gel-based softwares exist to detect protein changes potentially associated with disease. The data, however, are abundant with technical and structural complexities, making statistical analysis a difficult task. A particularly important topic is how the various softwares handle missing data. To date, no one has extensively studied the impact that interpolating missing data has on subsequent analysis of protein spots.

**Results:**

This work highlights the existing algorithms for handling missing data in two-dimensional gel analysis and performs a thorough comparison of the various algorithms and statistical tests on simulated and real datasets. For imputation methods, the best results in terms of root mean squared error are obtained using the least squares method of imputation along with the expectation maximization (EM) algorithm approach to estimate missing values with an array covariance structure. The bootstrapped versions of the statistical tests offer the most liberal option for determining protein spot significance while the generalized family wise error rate (gFWER) should be considered for controlling the multiple testing error.

**Conclusions:**

In summary, we advocate for a three-step statistical analysis of two-dimensional gel electrophoresis (2-DE) data with a data imputation step, choice of statistical test, and lastly an error control method in light of multiple testing. When determining the choice of statistical test, it is worth considering whether the protein spots will be subjected to mass spectrometry. If this is the case a more liberal test such as the percentile-based bootstrap *t *can be employed. For error control in electrophoresis experiments, we advocate that gFWER be controlled for multiple testing rather than the false discovery rate.

## Background

Analysis of quantitative changes in a specific proteome (i.e., complement of proteins expressed in a particular tissue or cell at a given time) is commonly carried out using two-dimensional gel electrophoresis (2-DE). With this procedure, proteins are separated in the first dimension based on iso-electric point, followed by separation based on molecular mass in the second dimension. Subsequently, protein spots are visualized, and the scanned gel images are analyzed using image analysis programs (e.g. ImageMaster, PDQuest). Once the relevant proteins spots have been determined, these specific proteins are identified using mass spectrometry. Because quantitative protein changes can be analyzed on a large scale, 2-DE frequently is used as an initial screening procedure whereby results obtained generate new hypotheses and determine the direction of subsequent studies. 2-DE analyses, however, are expensive and can be time-consuming; these issues result in a possibly limited sample size. Furthermore, in some cases (e.g., aging studies, chronic drug treatment, screening for biomarker) replication of the study may be prohibitive. The above factors not only make it critically important to correctly analyze the 2-DE results, but also to maximize information obtained.

The statistical analysis of 2-DE gels can be divided into two classes: analysis via spot finding, and analysis using image modeling and decomposition such as described in [1]. For our purposes, we will focus on the former 2-DE analysis, employing spot detection and spot matching across gels. In this analysis, a common problem is the presence of missing values. This generally occurs when a protein spot is not found on all gels. Missing spot values can be caused by technical issues such as variations in spot migration and staining, background noise or distortions in gel images, and the ability of the image analysis software to detect and match the protein spots across the gels. Values also may be missing, however, due to biological variation; here, the protein amount in some samples may fall below the detection limit, or post-translational modifications may alter the migration of the protein on the gel. It has been reported that 30% of data points may be missing in 2-DE analyses [2-4].

Besides the obvious loss of information due to missing values, data analysis is also hampered by missing values. Clustering techniques (e.g., *k*-means, hierarchical) and various statistical approaches (such as principal component analysis (PCA) and significance analysis of microarrays (SAM)) require complete datasets [3,5]. The prevalence of missing values in 2-DE and associated uncertainty as to the cause presents a dilemma on handling missing values. Some image analysis programs, including ImageMaster TM 2D Platinum, substitute missing values with zeroes which potentially could lead to an erroneous interpretation of the results if the values were missing for technical rather than biological reasons [6]. Omitting protein spots that contain missing values would result in a dramatic loss of information since a significant number of the protein spots will have missing values [2-4]. Replicating the study may likewise be impractical and would provide only a marginal advantage, given the prevalence of missing values. Running multiple gels for each sample and then using a composite gel in subsequent statistical analyses will reduce variability due to technical issues and also might reduce the number of missing values caused by non-biological reasons (e.g., image analysis software). Running replicate samples, however, will lead to a proportional increase in the total number of gels to be run, and the logistics of running these additional gels will likely strain resources; this can cause fewer samples to be analyzed. Because technical replication is less beneficial than biological replication in reducing variability, the former should not be pursued at the expense of the latter [7].

A solution to the problem of missing values is to "impute" these data, i.e. replace the missing spot values with values that use information from the protein spots that are present. Various imputation methods have been applied to microarray data, thereby improving detection of differentially expressed genes (e.g., [8-16]). Several works have, likewise, extensively compared these methods on microarray data [17-19]. In contrast, however, data imputation has found less extensive use in proteomic studies with little work comparing such approaches for proteomic data [2,4,20,21].

This study compares various imputation methods (and studies their impact on typically-used high-level statistical methods) in 2-DE studies. We examine two datasets for this study. The first is an unpublished dataset from Dr. Rabin's laboratory (Rabin dataset), comparing a control condition against phorbol 12-myristate 13-acetate (PMA; see Methods). The second dataset (Coling dataset) was developed to analyze cisplatin-induced cochlear damage, see [22]. We assume that the image processing has been suitably performed, including the spot matching across gels. Our starting point for analysis is the data matrix with rows corresponding to spots and columns corresponding to gels. The (*i*, *j*)th entry in the matrix represents the normalized spot volume for the *i*th spot from the *j*th gel. Note the similarity between this "proteomic matrix" and the "gene expression matrix" which is a common starting point in microarray analysis. For our analysis, we focus on two main areas: the influence of different imputation methods, and the influence of different statistical tests in determining what protein spots are present in different amounts between two conditions. We examine four different imputation methods and six different statistical tests on both real and simulated datasets. The imputation methods considered are the row average (RA) method, the *k *nearest neighbors (KNN) method, the least squares method (LSM), and nonlinear partial least squares (NIPALS) method. The statistical tests under consideration are the parametric *t *test, permutation *t *test, the "Chebby Checker" test, and three different types of bootstrap tests. All of the imputation methods and statistical tests are further detailed in the Data Analysis section. To compare the methods, we randomly remove data points from the datasets and compare the results between the complete dataset and the dataset(s) with simulated missing spots.

## Results & Discussion

The PMA-treated gel that was used as the reference gel in the Rabin dataset is shown in the Additional Materials (Additional file 1, Figure S1). The Deleted Residuals method in HDBStat! was used to test for non-homogeneity among the gels and to identify gels that are outliers in comparison with other gels in the group [23]. No gels were determined to be outliers via this method (results not shown).

**Figure 1:**
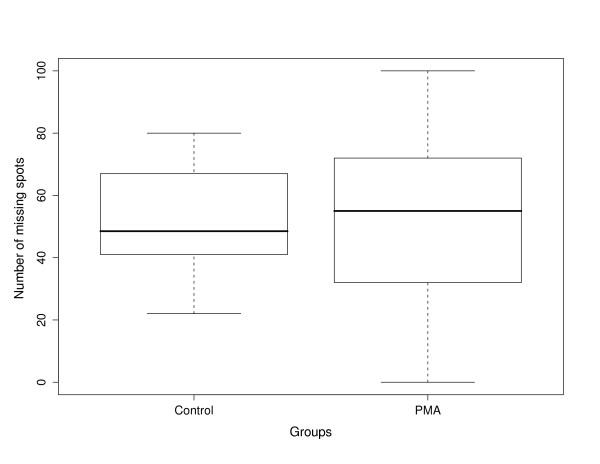
**Comparison of the number of missing protein spots in the 2-D gels obtained from the control and PMA-treated groups, respectively**. Spots were defined as missing if they appeared in the reference gel, but were not found in the other gels. Mean number of missing spots in the control and PMA-treated groups was 51.2 ± 8.3 and 52.3 ± 14.1, respectively (*N *= 6).

As shown in Figure 1 for the Rabin dataset, there was no statistically significant difference in means between the treatment groups with respect to the mean number of missing protein spots in each gel (*p *= 0.945). For both groups in the Rabin dataset, the number of complete spots (i.e., protein spots found in all six gels from control or PMA-treated samples) increased as spot intensity increased (Additional file 2, Figure S2). Thus, it appears that there is an association between missing values and fluorescent intensity of the protein spot, i.e. missing values are more likely to occur in proteins with lower intensity. There did not appear to be, however, any relationship between the absence of a protein spot and its location on the gel; missing spots were randomly distributed across the gel with no observable bias with respect to their gel location (results not shown). The following subsections detail the results for the Rabin dataset. Similar results for the Coling dataset can be found in the Additional Materials.

### Imputation Results

**Figure 2:**
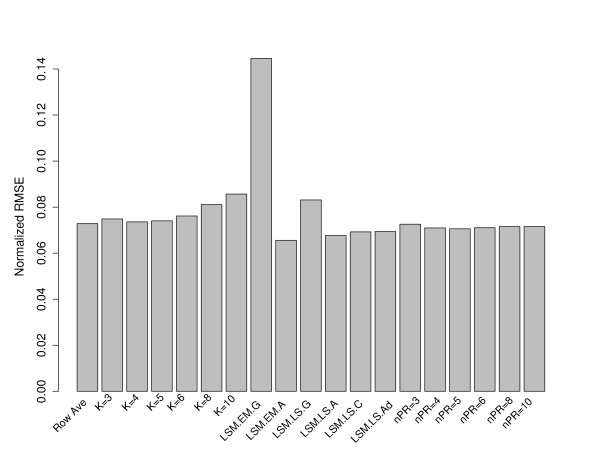
**Comparison of *k *nearest neighbor (KNN), Row Average, and Least Squares Methods (LSM), and NIPALS imputation methods**. 500 simulations were performed, where each simulation generated a dataset containing 20% missing values by randomly removing spot values from the complete matrix of 70 protein spots. Missing values were imputed using row average (Row Ave), the KNN method with different k nearest neighbor values, or LSM method and the results compared using the normalized root mean square error (RMSE). One set of LSM options allow the user to choose a correlation between protein spots estimated via least squares (LSM.LS.G) or via the EM algorithm (LSM.EM.G). Another set of LSM options allows the user to choose a correlation between arrays estimated via least squares (LSM.LS.A) or via the EM algorithm (LSM.EM.A). The user is allowed a combined (array and spot) correlation (LSM.LS.C) and adaptive (LSM.LS.Ad) correlation procedure. The NIPALS methods are summarized by "nPR" which denotes the number of principal components used to impute the missing data.

In Figure 2, for the Rabin Dataset (Coling Dataset: Additional file 3, Figure S3), we examined the mean RMSE when 20% of the data is randomly removed (500 times) from the complete dataset. From Figure 2, the RA method was comparable to the KNN imputation with *k *= 4 or *k *= 5, however, the LSM method "EMimpute_array" (LSM.EM.A) performed the best among the imputation procedures, as demonstrated with the smallest RMSE value. This option uses an expectation-maximization (EM) approach to estimate missing values with an array covariance structure. The EM algorithm in this case iteratively updates the estimates of the covariance matrix and missing values. As expected the "EMimpute_gene" (LSM.EM.G) and "LSimpute_gene" (LSM.LS.G) performed unfavorably in terms of RMSE. These methods use a weighted average of several single regression estimates of the same missing value where for the missing spot *y*, the *k *nearest (correlation wise) spots are included in the prediction model and none of the *k *nearest spots are allowed to have missing values in the same gel as the missing value to be estimated. In this algorithm, the number of nearest spots is fixed at 10. With only 70 protein spots under consideration in the Rabin dataset, it is most likely the case that the 10 "nearest" spots, in fact, show little correlation with the protein spot containing the missing data.

Studying the KNN methods in Figure 2, we can see the sensitivity associated with the choice of *k *in the KNN imputation method. In general, the mean RMSE increases as *k *increases. This is expected since large values of *k *tend to over-fit the data, hence leading to a large RMSE when applied to the missing data; meanwhile, small values of *k *lead to simpler models that will likely fit the missing data better in terms of a smaller RMSE. We further see that small values of *k *(*k *< 6) showed similar performance in terms of RMSE.

From Figure 2, we see that the NIPALS method performs favorably compared to KNN, and also there appears to be minimal dependence on RMSE and the number of principal components employed in the NIPALS procedure.

**Figure 3:**
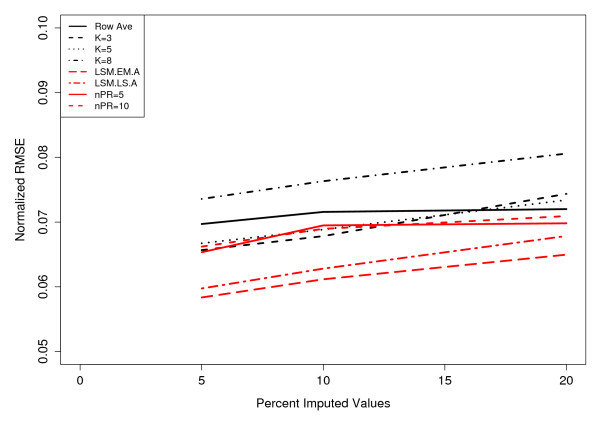
**Effects of the amounts of missing data on imputation procedures**. 500 simulations were performed, where each simulation generated a datasets containing 5%, 10%, and 20% missing values by randomly removing spot values from the complete data set of 70 protein spots. Missing values were imputed by row average (Row Ave), LSM, KNN, and NIPALS imputation methods with *k *nearest neighbor values of 3, 5, or 8. Results of the imputation were compared using RMSE. The NIPALS methods are summarized by "nPR" which denotes the number of principal components used to impute the missing data.

Based on the results from Figure 2, we restrict the LSM options to only the "EMimpute_array" (LSM.EM.A) and "LSimpute_array" (LSM.LS.A) choices, since these options yield the smallest mean RMSE with 20% of the data removed. Since all NIPALS methods perform equally well in Figure 2, we choose to examine the results when only using 5 or 10 principal components. In Figure 3 (Coling Data: Additional file 4, Figure S4), we examined the RMSE for different methods of imputation, as a function of the percentage of missing data (5%, 10%, or 20%) from the complete dataset. All methods of imputation show a positive correlation with the percentage of missing data. As suggested by the results in Figure 3, the LSM option with "EMimpute_array" has the smallest RMSE regardless of the percentage of missing data. Also, the KNN method with *k *= 8 neighbors has the largest RMSE regardless of the percentage of imputed data. Roughly speaking (outside of the RA method), each method has the same correlation (slope) between RMSE and percentage of imputed data.

**Figure 4:**
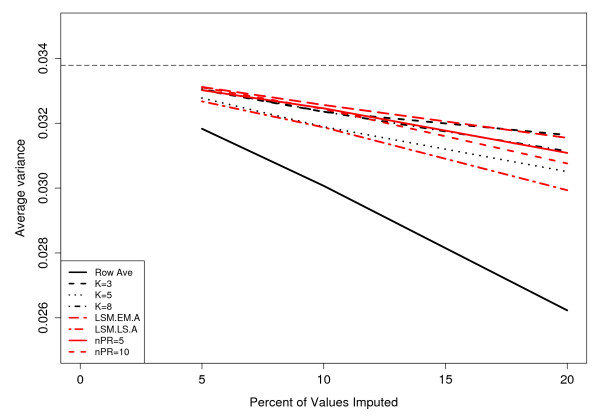
**Effects of imputation on the average variance**. 500 simulations were performed, where each simulation generated a dataset that contained 5%, 10%, and 20% missing-ness by randomly removing spot values from the complete dataset. Missing values were imputed by row average (Row Ave), LSM, and NIPALS or *k *nearest neighbors (KNN) imputation with *k *= 3, 5 or 8. Average variances of the complete 70 protein spots without missing data (horizontal dotted-dashed line) and after imputation are shown. The NIPALS methods are summarized by "nPR" which denotes the number of principal components used to impute the missing data.

In addition to examining the correlation between RMSE and percentage of imputed data, Figure 4 examines the average variance as a function of the percentage of data imputed for the Rabin Dataset (Coling Dataset: Additional file 5, Figure S5). In this setting, the average variance is the mean of the variance of each spot in the imputed dataset. Since all imputation methods imputed missing data by using a summary score based on the available data, the imputation methods produce average variances less than the complete (original) dataset. Also (as expected), the average variance significantly decreases as a function of the percentage of data imputed. The RA method yields an average variance considerably less than all other imputation methods. Broadly speaking, outside of the RA method, the imputation procedures were very similar in terms of average variance.

### Statistical Test Results

The effects of imputation on the subsequent statistical analysis using the parametric *t *test (unequal variances), permutation *t *test, bootstrap *t *test(s), and Chebyshev's inequality test ("Chebby Checker") were investigated. For these studies, the statistical analysis of either dataset (Rabin or Coling) was compared to the analysis using the dataset and simulating 10% randomly missing values which were then imputed using the RA, KNN (with *k *= 1 and 5), and LSM methods.

**Figure 5:**
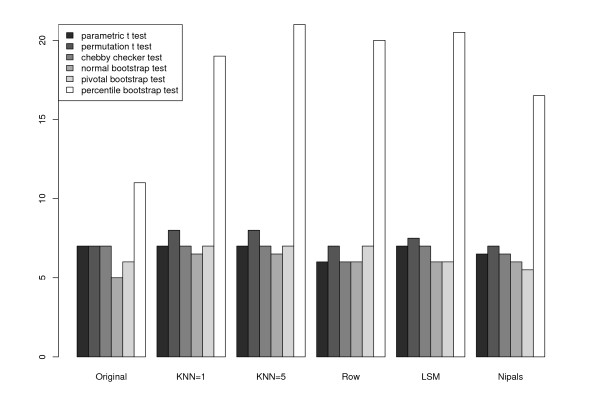
**Median number of spots discovered using each method compared against the number of discoveries on the complete data**. Randomly 10% of the data was removed and imputed using each method and test for significance was a *p*-value < 0.05. For the NIPALS methods five principal components were used to impute the missing data.

**Figure 6:**
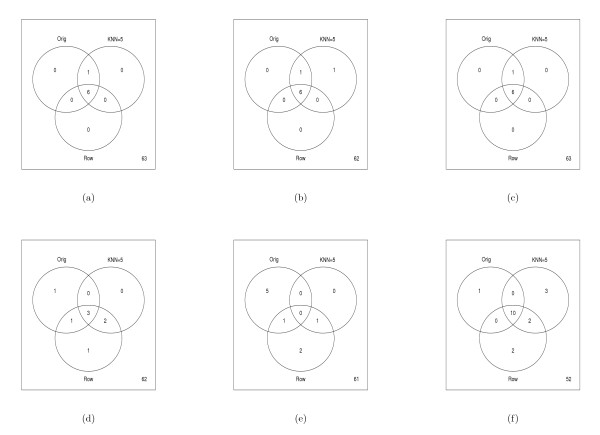
**Summary of Significant Spots on Rabin Dataset: Multiple (20) datasets containing 10% missing values were generated by randomly removing spots values from the complete (Orig) dataset containing 70 proteins**. Missing values were then imputed using the RA, LSM, or KNN method with *k *= 5. Values in the Venn diagrams represent the number of discovered proteins (*p*-value < 0.05) in the original complete dataset and the imputed datasets. Note, to be discovered in KNN method or Row Average method for the imputed datasets, the spot needed to have a *p*-value less than 0.05 in at least half of the simulated datasets. The Venn diagrams refer to (a) parametric *t *test, (b) permutation *t *test, (c) Chebby Checker test, (d) normal-based bootstrap *t *test, (e) pivotal-based bootstrap *t *test, (f) percentile-based bootstrap *t *test.

**Figure 7:**
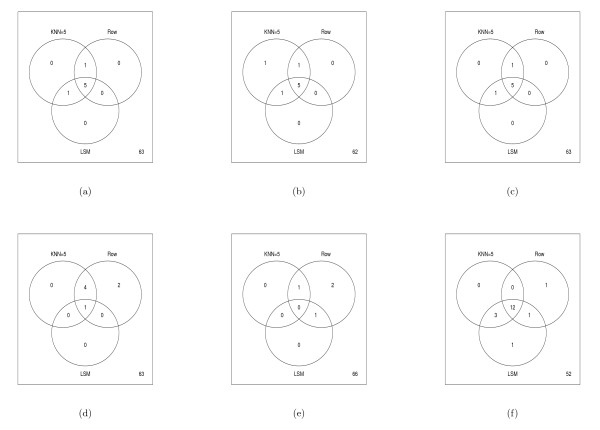
**Summary of Significant Spots on Rabin Dataset: Multiple (20) datasets containing 10% missing values were generated by randomly removing spots values from the complete dataset containing 70 proteins**. Missing values were then imputed using the Row Average method or KNN method with *k *= 5. Values in the Venn diagrams represent the number of discovered proteins (*p*-value < 0.05) in the original complete dataset and the imputed datasets. Note, to be discovered in KNN, RA, or LSM method for the imputed datasets, the spot needed to have a *p*-value less than 0.05 in at least half of the simulated datasets. The Venn diagrams refer to (a) parametric *t *test, (b) permutation *t *test, (c) Chebby Checker test, (d) normal-based bootstrap *t *test, (e) pivotal-based bootstrap *t *test, (f) percentile-based bootstrap *t *test.

For the datasets, the parametric *t*, permutation *t*, and Chebyshev's inequality tests performed similarly in identifying protein spots that were likely to be altered by the PMA treatment, while the percentile bootstrap appeared more liberal (Figure 5). To quantify the statistical tests in light of imputation procedures, we removed 10% of the data values from the complete dataset and examined which proteins were identified as being changed in the imputed dataset as well as the total number of differentially altered proteins in the imputed datasets. For robustness of these results, we repeated the process of removing 10% of the data 20 times. Figure 5 shows the median number of spots discovered over the 20 simulations for the Rabin Dataset (Coling Dataset: Additional file 6, Figure S6). The results are also summarized via Venn diagrams (Figures 6 and 7, Coling Dataset: Additional file 7, Figure S7 and Additional file 8, Figure S8). From Figure 6 (Coling Dataset: Additional file 7, Figure S7), with 10% of the data removed within all tests there was still a fairly large agreement between imputation methods and the complete dataset. Comparing the different tests, we see that the parametric *t*, permutation *t*, and Chebyshev's inequality tests were very similar in terms of robustness to imputation methods. The bootstrap-based tests (Figures 6(d), (e), and 6(f)) showed varying degrees of agreement across imputation methods. Specifically, with the normal based bootstrap *t *test, one spot was found in the complete dataset but in neither the KNN or RA imputed datasets, while five spots were missed with the pivotal-based bootstrap *t *test. Also, the percentile-based bootstrap *t *test demonstrated the most liberal number of discoveries in the complete dataset, as well as the imputed datasets. In the percentile-based bootstrap *t *test, ten spots were found in common in the complete Rabin dataset and the KNN and RA imputation methods. Figure 7 (Coling Dataset: Additional file 8, Figure S8) shows the agreement between the KNN, RA, and LSM imputation methods on the complete dataset with multiple (20) simulations of 10% missing data at random in the dataset. All three imputation methods have fairly good agreement, where again, the percentile-based bootstrap *t *test had the most discoveries, with 12 spots found in at least half of the simulated datasets regardless of imputation procedure.

In short, our conclusions regarding the statistical tests likewise hold for the Coling dataset. The percentile bootstrap test yields the largest number of mean discoveries (Additional file 6, Figure S6), while the most conservative tests are the normal bootstrap test and pivotal bootstrap test. These differences between the bootstrap tests are due to the nature of the bootstrap assumptions regarding the distribution of the test statistics [24]. In both datasets, the parametric *t*, permutation *t*, and "Chebby Checker" tests yield an intermediate number of discoveries compared to the bootstrap testing methods.

## Discussion

In the analysis of the Rabin dataset, approximately 60% of protein spots had at least one missing data point (i.e., a spot not observed in at least one gel). This resulted in a proteomic "expression" matrix that contained 21% missing values. Similar studies using 2-DE analysis have reported at least 30% missingness in their data [2-4]. In this study, the presence of a missing value was not related to the PMA treatment. Further, missing values were not dependent on location of the spot on the gel; this indicates the absence of bias based on isoelectric point or molecular weight. More missing values were found, however, in the bottom quartile of spot intensities, while the top quartile had the most number of complete protein spots. This indicates an association between missing values and the overall abundance of a protein (results not shown). Similarly, [25] reported that, as spot volume increased, the number of matched spots also increased. It is unclear whether the greater amount of missing values in the bottom quartile of spot intensities was related to biological variation or the technical difficulty of the software to detect, align, and match spots of low intensity. The ImageMaster TM 2D Platinum software (Version 5.0) clearly does better with the high intensity protein spots, although it may be worthwhile to consider normalization methods (e.g., [26]) in conjunction with image analysis software.

The performance of all the imputation methods, however, depended on the fraction of values that were missing. For the RA, the increase in RMSE was directly related to the percent of missing values. Lower percentages showed similar average variances between KNN, RA, and LSM imputation schemes. As the percentage of imputed values increased, however, the average variance of KNN and LSM imputation methods decreased slightly, whereas the variance with RA had a more profound decrease. A decrease in variance after RA imputation would increase the number of protein spots that would be identified as significantly altered thereby potentially inflating the occurrence of false positives. Based upon our findings, one should restrict the maximal fraction of values that are imputed in 2-DE studies. To accomplish this, we suggest only analyzing protein spots that are present in a majority of the gels. The criterion for a majority of spots to be present will reduce the loss of information due to missing values, while limiting the amount of required imputation. In addition, greater confidence in imputation accuracy may be obtained as the imputed values can be checked against the actual spot values observed.

The ability to detect differentially expressed proteins with 2-DE depends not only on the method and level of imputation, but also on the statistical analysis. This study applied six univariate tests to evaluate differences in protein amounts. While powerful, parametric methods require a number of assumptions, including that the data represent random samples from a Gaussian distribution; that may be difficult to assess due to the small sample size typically used in proteomic studies. An inflated Type I error can occur, for example, if the data do not fit a normal distribution [27]. Permutation tests, which generate their own distribution and do not make any assumptions about the underlying distribution of the test statistic [28-31], have been suggested to be more powerful than parametric tests and should be preferred for small sample sizes [32,33]. The "Chebby Checker" variation of Chebyshev's inequality test is robust against departures from normality and inequality of variance in small datasets [34]. The bootstrap *t *test is useful with data that does not conform to known statistical distributions. The bootstrap methods, however, cannot completely alleviate the difficulties caused by a small sample size. With only six gels per treatment group, we restricted our bootstrap simulations and only used 25 bootstrap resampled datasets.

Of the statistical methods used, the percentile-based bootstrap *t *test was the most liberal in detecting differentially expressed proteins, while the normal-based bootstrap *t *test appeared to be the most conservative and thus potentially the least sensitive. The parametric *t*, permutation *t*, and Chebby Checker tests yielded comparable results and displayed an intermediate amount of discovered proteins. While these relationships between statistical tests were observed irrespective of the imputation method used, the imputation method slightly impacted the selection process (statistical tests) identifying "changed proteins".

## Conclusions

The salient question is how best to analyze the results of 2-DE analysis. This issue is complicated by the fact that the statistical analysis involves the testing of a large number of hypotheses and is performed without knowledge of the identity of the proteins involved. In a typical quantitative proteomic study using 2-DE, statistical analyses are used to determine which protein spots are differentially expressed and subsequently will be subjected to mass spectrometry for protein identification. Thus, unlike analysis of microarray data, statistical analysis of 2-DE data occurs without the possible benefit of relevant biological information (e.g., cellular function of the protein, or how it is regulated) that may help either to substantiate the statistical analysis or to identify possible false positives. As the intent of the statistical analysis is to provide an objective means of identifying which proteins are changed and thus allowing the proteins to be prioritized (i.e. "triaged") for subsequent study, the statistical analysis should identify as many true effects as possible while incurring few or at least a low proportion of false positives. Specifically, the statistical methods used to analyze 2-DE data should be guided primarily by the study objective and whether making a Type I or a Type II error is more egregious. For example, if 2-DE analysis is being used in an initial screening procedure to identify candidate proteins as possible biomarkers, greater concern at first might be with omitting a true effect as false positives would be weeded out in subsequent studies. In summary, we advocate for a three-step statistical analysis of 2-DE data with a data imputation step, choice of statistical test, and lastly an error control method in light of multiple testing. For imputation methods, the best results in terms of RMSE are obtained using the LSM imputation method with the EM algorithm approach to estimate missing values with an array covariance structure. When determining the choice of statistical test, it is worth considering whether the protein spots will be subjected to mass spectrometry. If this is the case, a more liberal test such as the percentile-based bootstrap *t *should be employed. Otherwise, outside of the bootstrap-based *t *tests, there are only relatively small differences between the different statistical tests. Specifically, the normal bootstrap test and the pivotal bootstrap test yield the smallest number of discoveries on both datasets, while the parametric and permutation *t *tests are in the middle in terms of number of discoveries. Lastly, for error control in testing protein spots in electrophoresis (e.g. usually < 1000 tests), from our work in [35], we advocate that gFWER be controlled rather than the false discovery rate.

## Methods

### Cell Culture

For the Rabin dataset, PC12 cells were cultured as previously described [36]. The cells were harvested and treated for 10 minutes at 34°C in PBS containing 1 *μ*M phorbol 12-myristate 13-acetate (PMA). Cells were collected by centrifugation at 14,000 g for 1 minute at 4°C, and the resulting cell pellet was resuspended in 50 mM Tris buffer (pH 7.4) containing complete EDTA-free protease inhibitors (Roche, Indianapolis, IN) and a cocktail of phosphatase inhibitors (1 mM Na_3_VO_4_, 2.5 mM sodium pyrophosphate, 1 mM *β*-glycerolphosphate). Samples were sonicated (three 5s bursts separated by one minute incubation on ice between each burst) and centrifuged at 40,000 g for 15 minutes at 4°C. The resulting supernatants were extracted with chloroform:methanol:water (1:4:3), and the proteins subsequently were precipitated with cold methanol. The resulting protein pellets were air-dried and then resuspended in 2X sample buffer (7 M urea, 2 M thiourea, 4% (w/v) CHAPS, 2% (v/v) IPG buffer 4-7, and 2% (w/v) dithiothreitol DTT), and then diluted with an equal volume of 2X rehydration buffer (5 M urea, 2 M thiourea, 4% (w/v) CHAPS, 0.002% (w/v) bromophenol blue, 20% (v/v) isopropanol, 10% (v/v) glycerol, 1% IPG buffer 4-7, and 2.8 mg/ml DTT) to yield a protein concentration of 1 *μ*g/*μ*l. The details on the cell cultures for the Coling dataset can be found in [22].

### 2-DE

For the Rabin dataset, isoelectric focusing (IEF) was carried out using an Ettan IPGphor (GE Healthcare) and 24 cm linear, pH 4-7 Immobiline DryStrips (GE Healthcare). The conditions used for IEF were: rehydration loading of the IPG strips at 30v for 12 hours, 500v for 1 hour, 1000v for 1 hour and 8000v for 8.20 hours. Subsequently, the IPG strips were incubated successively (15 minutes each) at room temperature in an equilibration buffer containing 50 mM Tris-HCl, 6 M urea, 30% (v/v) glycerol, 2% (w/v) SDS, 0.002% (w/v) bromophenol blue and 2% (w/v) DTT (pH 8.8) followed by an incubation in the above buffer, but with 4.5% (w/v) iodoacetamide in place of DTT. Electrophoresis was carried out using an Ettan DALTsix Electrophoresis System (GE Healthcare) and 1 mm thick 10% SDS-PAGE (25.5 cm × 20.5 cm) gels. Six controls and six PMA-treated samples were separated. To ensure that gels remained attached to the plates during scanning and spot picking, the plates were pre-coated with Bind-Silane (GE Healthcare). In addition, self-adhesive markers were also placed on the plates coated with Bind-Silane to facilitate spot localization by an Ettan DALT spot picker (GE Healthcare). The conditions for electrophoresis were 5 W/gel for 30 min followed by 10 W/gel until the bromophenol blue dye front was approximately 1 mm from the bottom of the plate. The gels were fixed and stained with ProQ Diamond^® ^(Molecular Probes) according to the manufacturer's instructions. The details on the 2-DE separation methods for the Coling dataset can be found in [22].

### Image analysis

For the Rabin dataset, fluorescent images of gels stained with ProQ Diamond^® ^(Molecular Probes) were acquired using a Typhoon 9400 variable mode imager (GE Healthcare). The scanned fluorescent images of ProQ Diamond^® ^were then analyzed using the ImageMaster TM 2D Platinum software (Version 5.0). To reduce variations due to manual cropping, the gel images were first cropped with Picture Manager (Microsoft) [36]. The following spot detection parameters were used for image analysis: Smooth 3, Minimum Area 5, and Saliency 6. An automatic spot detection algorithm was used, and manual editing of spots was avoided in the analysis to minimize quantitation errors. One of the PMA-treated gels was used as the reference gel for spot matching and alignment. Spot volumes were normalized using the mean-normalization method (i.e. spot volume for a specific protein spot was divided by the spot volumes for all the spots in the gel). Spot normalization reduces experimental variations between gels caused by conditions such as differences in protein loading or staining. The details on the 2-DE imaging and normalization methods for the Coling dataset can be found in [22]. For the analysis of the Coling dataset, we used the Cy2 channel to normalize the Cy3 and Cy5 channels.

### Data Analysis

The design for the Rabin dataset consisted of six gels for each of two conditions, a control condition and the PMA condition. After image analysis, a data matrix consisting of normalized spot volumes was produced where the rows corresponded to spots and the columns corresponded to gels.

The Rabin dataset includes the 70 protein spots that were matched or found in the all of the gels. In the Additional Materials, we included the analysis of the Coling dataset, where the Coling dataset contains 343 protein spots that were found in all gels in the analysis. For each of the datasets, the three main steps in the analysis pipeline were 1) imputation of missing data 2) statistical testing and 3) error control in light of multiple testing.

For either the Rabin or Coling dataset (see Additional Materials), four different imputation procedures were performed, the Row Average (RA), the *k *nearest neighbor (KNN), nonlinear iterative partial least squares (NIPALS), and least square method (LSM) [37]. The Row Average (RA) and *k *nearest neighbor (KNN) imputation were done using the *R *computing language with the *impute *package [38] while LSM was implemented using the java language code [39]. In the RA method, the average of the values that are present for that particular protein spot are used to replace the missing data points. The KNN algorithm classifies objects based on closest ("nearest") protein spots. In this algorithm we find the *k *nearest neighbors using a suitable distance metric, and then we impute the missing elements by averaging those (non-missing) elements of its neighbors. In the KNN method, there are different types of distance metrics (Pearson correlation, Euclidean, Mahalonobis, and Chebyshev's distance) that can be employed. We chose the Euclidean distance metric as it has been reported to be more accurate [17]. Although designed for microarray data, we have employed the LSM method to our proteomic dataset.

The NIPALS method is summarized in [40] and is implemented using the *R *package "pcaMethods" [41]. Similar to KNN, in order to implement the NIPALS algorithm, it is necessary for the user to specify the number of principal components.

The LSM method estimates missing values utilizing correlations between protein spots and gels. There are several variants of the LSM described fully in [37], where each variation is related to different methods of estimating the correlation within the dataset. The LSM method was implemented using the LSimpute.jar java script available at http://www.ii.uib.no/~trondb/imputation/. To evaluate the three different methods of imputation, spot values were randomly deleted across groups from the complete dataset, and the normalized root mean square error (RMSE) was calculated to compare the imputation methods.

The second step in the pipeline is to employ a statistical test on each protein spot to assess whether the spot is present in different amounts between the conditions. For this analysis, six different statistical tests were examined, specifically, the standard *t *test, Chebyshev's inequality test, permutation *t *test and three different variants of the bootstrap *t *test (normal approximation, percentile, and pivotal). The permutation *t *test was performed using the *Deducer *software package in *R *[42]. The standard *t *test (unequal variances) and Chebyshev's inequality test were carried out using standard *R *functions. The version of Chebyshev's inequality test (or "Chebby Checker") is described in Equation (7) in [34]. There are three types of bootstrap tests that can be performed: tests derived from the normal approximation, percentile confidence intervals, and pivotal confidence intervals [43]. For the class of bootstrap tests, the confidence intervals were inverted in order to obtain the p values for each protein spot. The output using all three bootstrapping methods are summarized in the Results.

After employing a statistical test for each protein spot, the third step in the pipeline is to determine protein spot significance with consideration of error control in light of multiple testing. To compare the number of significant spots across different simulated imputation procedures from the complete dataset, the per comparison error rate was controlled at 0.05. To examine imputation methods, 20 different Monte-Carlo simulations were performed, where each simulation consisted of randomly deleting 10% of the data from the complete dataset and imputing the data using either KNN, RA, or LSM imputation. We summarized the imputation methods using spots where the *p*-value for significance was less than 0.05 in at least half of the simulated datasets. We recognize that controlling the per comparison error rate is likely to inflate the number of false positives, nevertheless, this method is acceptable for comparing imputation procedures since we are not making claims that the discovered spots are truly differentially expressed.

In practice, when determining protein significance, from our work in [35], we advocate controlling the generalized family wise error rate (gFWER). An overview of methods to control gFWER is available in [44] with their implementation in the *R *software provided in the package *multtest *[45].

## List of Abbreviations

2-DE: two-dimensional gel electrophoresis; EM: expectation maximization; FDR: false discovery rate; gFWER: generalized family wise error rate; IEF: isoelectric focusing; KNN: *k *nearest neighbor; LSM: least square method; NIPALS: nonlinear partial least squares; PCA: principal component analysis; PMA: phorbol 12-myristate 13-acetate; RA: row average method; RMSE: root mean square error; SAM: significance analysis of microarrays

## Competing interests

The authors declare that they have no competing interests.

## Authors' contributions

JCM designed the study, performed the statistical analysis, and wrote the manuscript. SD designed the study, performed the data analysis, and wrote the manuscript. KFS assisted in the statistical analysis and the writing of the manuscript. RAR provided materials and contributed to the conception of the study. All authors read and approved the final manuscript.

## Supplementary Material

Additional file 1 **Figure S1**. **Rabin Dataset**: Representative image of a 2-D gel stained with ProQ Diamond. PC-12 cells were treated with PMA, and the proteins separated by 2-D gel electrophoresis as described in Methods. Figure shown was representative of the 2-D gels obtained from the six controls and six PMA-treated samples, and was used as the reference gel for image analysis. Click here for file

Additional file 2 **Figure S2**. **Rabin Dataset**: Frequency distribution of number of complete protein spots as function of fluorescent intensity (abundance) of the spot. The number of specific protein spots that appeared in all six gels from the control or PMA-treated samples is plotted as a quartile frequency distribution of the average fluorescent spot intensities. Click here for file

Additional file 3 **Figure S3**. **Coling Dataset**: Comparison of *k *nearest neighbor (KNN), Row Average, Least Squares Methods (LSM), and NIPALS imputation methods on the dataset in [22]. 500 simulations were performed, where each simulation generated a dataset containing 20% missing values by randomly removing spot values from the complete matrix of 343 protein spots. Missing values were imputed using row average (Row Ave), the KNN method with different k nearest neighbor values, or LSM method and the results compared using the normalized root mean square error (RMSE). One set of LSM options allow the user to choose a correlation between protein spots estimated via least squares (LSM.LS.G) or via the EM algorithm (LSM.EM.G). Another set of LSM options allows the user to choose a correlation between arrays estimated via least squares (LSM.LS.A) or via the EM algorithm (LSM.EM.A). Lastly, the user is allowed a combined (array and spot) correlation (LSM.LS.C) and adaptive (LSM.LS.Ad) correlation procedure. The NIPALS methods are summarized by "nPR" which denotes the number of principal components used to impute the missing data. Click here for file

Additional file 4 **Figure S4**. **Coling Dataset**: Effects of the amounts of missing data on imputation using Row Average, Least Squares Methods (LSM), KNN and NIPALS imputations methods in the dataset in [22]. 500 simulations were performed, where each simulation generated a datasets containing 5%, 10%, and 20% missing values by randomly removing spot values from the complete data set of 343 protein spots. Missing values were imputed by row average (Row Ave), LSM, and KNN methods with *k *= 4. The NIPALS methods are summarized by "nPR" which denotes the number of principal components used to impute the missing data. Results of the imputation were compared using RMSE. Click here for file

Additional file 5 **Figure S5**. **Coling Dataset**: Effects of imputation on the average variance for the dataset in [22]. 500 simulations were performed, where each simulation generated a dataset that contained 5%, 10%, and 20% missing-ness by randomly removing spot values from the complete dataset. Missing values were imputed by row average (Row Ave), LSM, or *k *nearest neighbors (KNN) imputation with *k *= 4. The NIPALS methods uses four principal components to impute the missing data. Average variances of the complete 343 protein spots without missing data (red horizontal dotted-dashed line) and after imputation are shown. Click here for file

Additional file 6 **Figure S6**. **Coling Dataset**: Median number of spots discovered using each method compared against the number of discoveries on the complete dataset in [22]. Randomly 10% of the data was removed and imputed using each method and test for significance was a *p*-value < 0.05. For the LSM method, we used the LSM option "EMimpute_array". For the NIPALS methods four principal components were used to impute the missing data. Click here for file

Additional file 7 **Figure S7**. **Coling Dataset**: Summary of Significant Spots on Coling Dataset: Multiple (20) datasets containing 10% missing values were generated by randomly removing spots values from the dataset containing 343 proteins. Missing values were then imputed using the RA, LSM ("EMimpute_array"), or KNN method with *k *= 5. Values in the Venn diagrams represent the number of discovered proteins (*p*-value < 0.05) in the original complete dataset and the imputed datasets. Note, to be discovered in KNN method or Row Average method for the imputed datasets, the spot needed to have a *p*-value less than 0.05 in at least half of the simulated datasets. The Venn diagrams refer to (a) parametric *t *test, (b) permutation *t *test, (c) Chebby Checker test, (d) normal-based bootstrap *t *test, (e) pivotal-based bootstrap *t *test, (f) percentile-based bootstrap *t *test. Click here for file

Additional file 8 **Figure S8**. **Coling Dataset**: Summary of Significant Spots on Coling Dataset: Multiple (20) datasets containing 10% missing values were generated by randomly removing spots values from the dataset containing 343 proteins. Missing values were then imputed using the Row Average method, LSM ("EMimpute_array"), or KNN method with *k *= 5. Values in the Venn diagrams represent the number of discovered proteins (*p*-value < 0.05) in the original complete dataset and the imputed datasets. Note, to be discovered in KNN, RA, or LSM method for the imputed datasets, the spot needed to have a *p*-value less than 0.05 in at least half of the simulated datasets. The Venn diagrams refer to (a) parametric t test, (b) permutation *t *test, (c) Chebby Checker test, (d) normal-based bootstrap *t *test, (e) pivotal-based bootstrap *t *test, (f) percentile-based bootstrap *t *test. Click here for file
