# A comparison of imputation procedures and statistical tests for the analysis of two-dimensional electrophoresis data

**DOI:** 10.1186/1477-5956-9-14

**Published:** 2011-03-29

**Authors:** Jeffrey C Miecznikowski, Senthilkumar Damodaran, Kimberly F Sellers, Donald E Coling, Richard Salvi, Richard A Rabin

**Affiliations:** 1 Department of Biostatistics, University at Buffalo, Buffalo NY 14214 USA; 2 Department of Pharmacology and Toxicology, School of Medicine and Biomedical Sciences, University at Buffalo, Buffalo NY 14214 USA; 3 Department of Mathematics and Statistics, Georgetown University, Washington DC 20057 USA; 4 Department of Communicative Disorders and Sciences, University at Buffalo, Buffalo NY 14214 USA; 5 Department of Biostatistics, Roswell Park Cancer Institute, Buffalo NY 14263 USA

## Correction

The list of authors of this article [1] was incorrect as published and should be as follows: Jeffrey C. Miecznikowski, Senthilkumar Damodaran, Kimberly F. Sellers, Donald E. Coling, Richard Salvi, Richard A. Rabin

Co-authors Donald E. Coling and Richard Salvi were omitted in error from the original published author list.

The Authors' contributions section should read:

JCM designed the study, performed the statistical analysis, and wrote the manuscript. SD designed the study, performed the data analysis, and wrote the manuscript. KFS assisted in the statistical analysis and the writing of the manuscript. DEC and RS provided the datasets for analysis. RAR provided the materials and contributed to the conception of the study. All authors read and approved the final manuscript.
