# Supplementary figures and images for: A comparison of imputation procedures and statistical tests for the analysis of two-dimensional electrophoresis data
Source: Proteome Sci. 2010 Dec 15;8:66. doi: 10.1186/1477-5956-8-66 (PMC3012036; doi:10.1186/1477-5956-8-66)

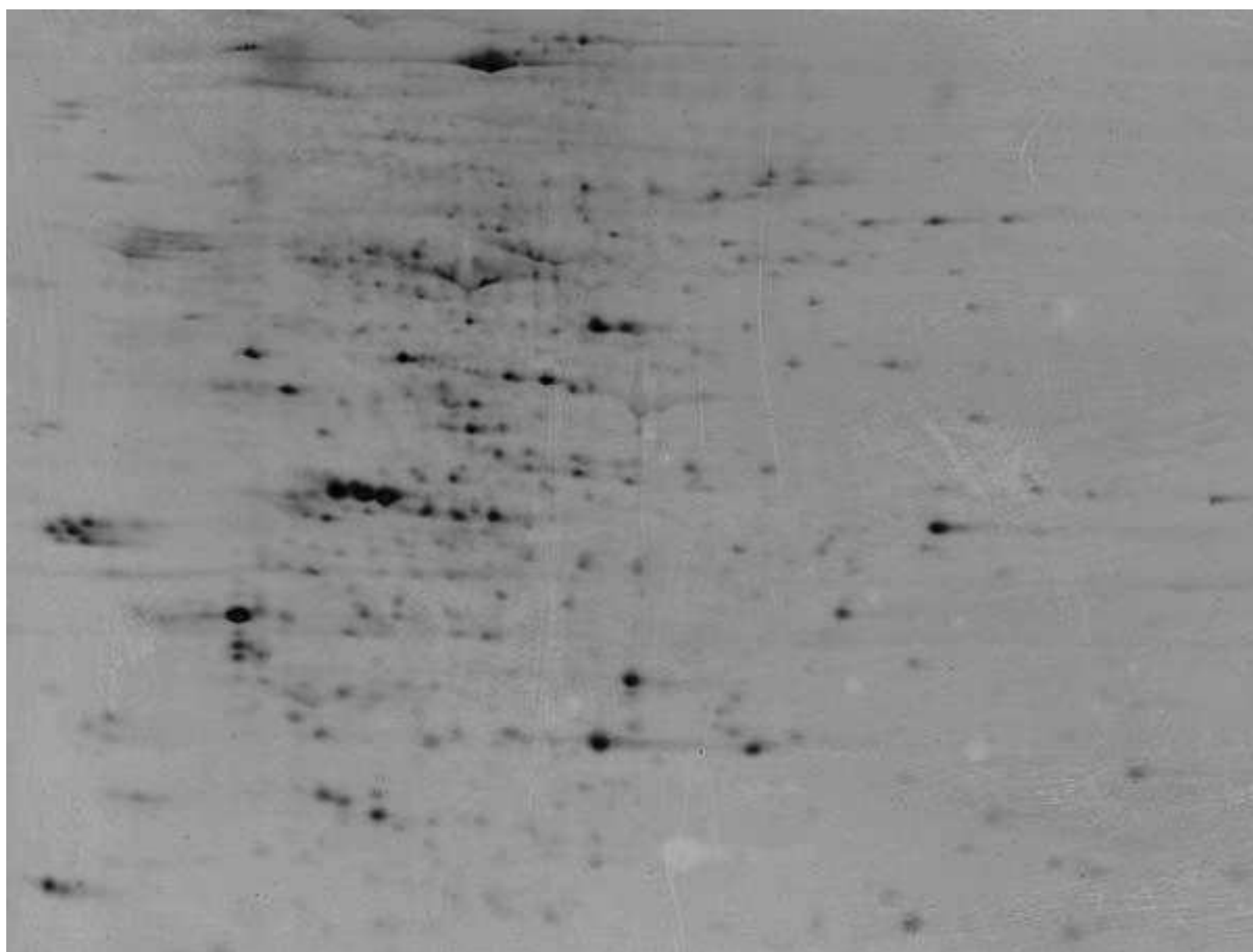

Supplement: Additional file 1 — Figure S1. Rabin Dataset: Representative image of a 2-D gel stained with ProQ Diamond. PC-12 cells were treated with PMA, and the proteins separated by 2-D gel electrophoresis as described in Methods. Figure shown was representative of the 2-D gels obtained from the six controls and six PMA-treated samples, and was used as the reference gel for image analysis. [file 1477-5956-8-66-S1.PDF]

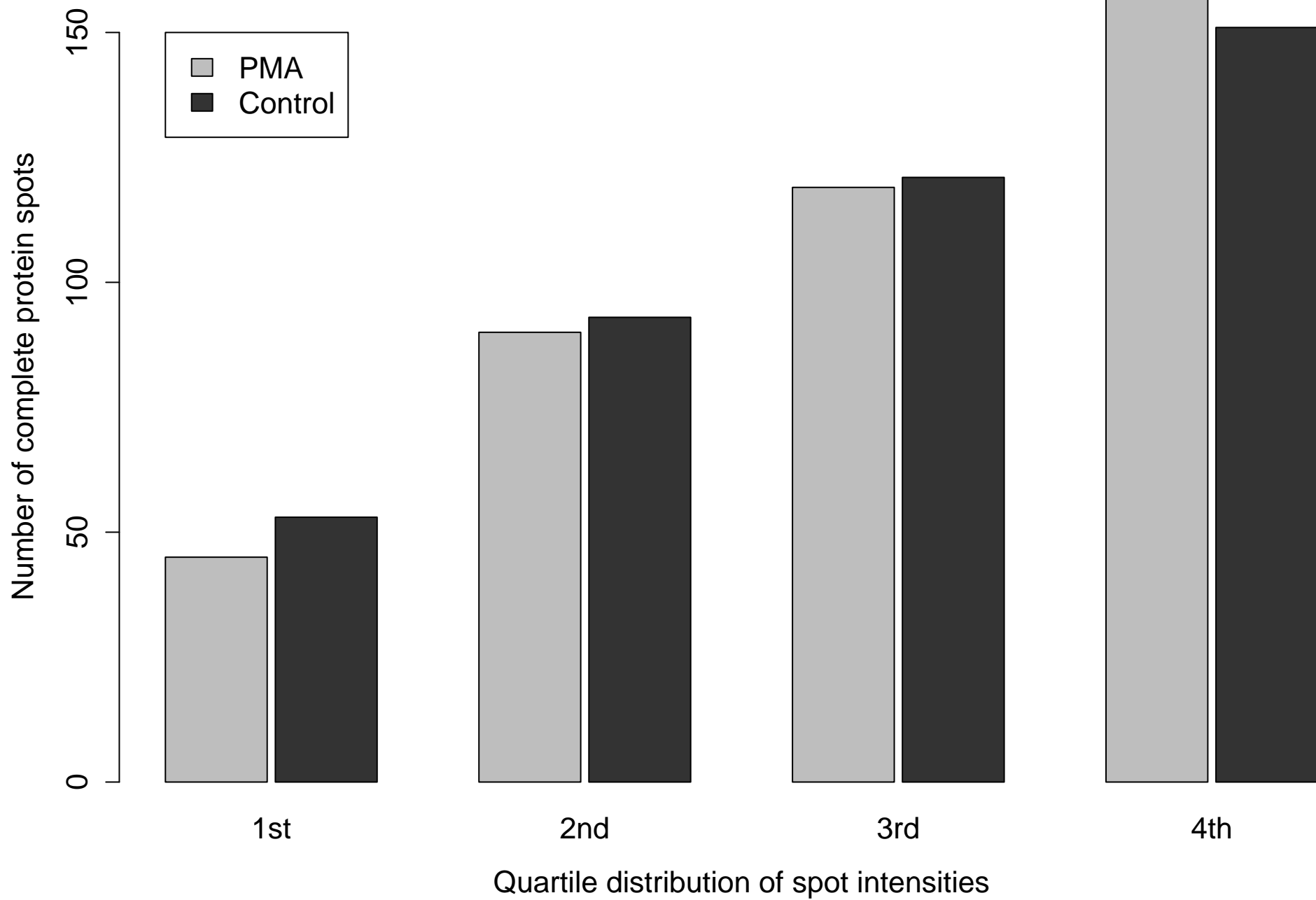

Supplement: Additional file 2 — Figure S2. Rabin Dataset: Frequency distribution of number of complete protein spots as function of fluorescent intensity (abundance) of the spot. The number of specific protein spots that appeared in all six gels from the control or PMA-treated samples is plotted as a quartile frequency distribution of the average fluorescent spot intensities. [file 1477-5956-8-66-S2.PDF]

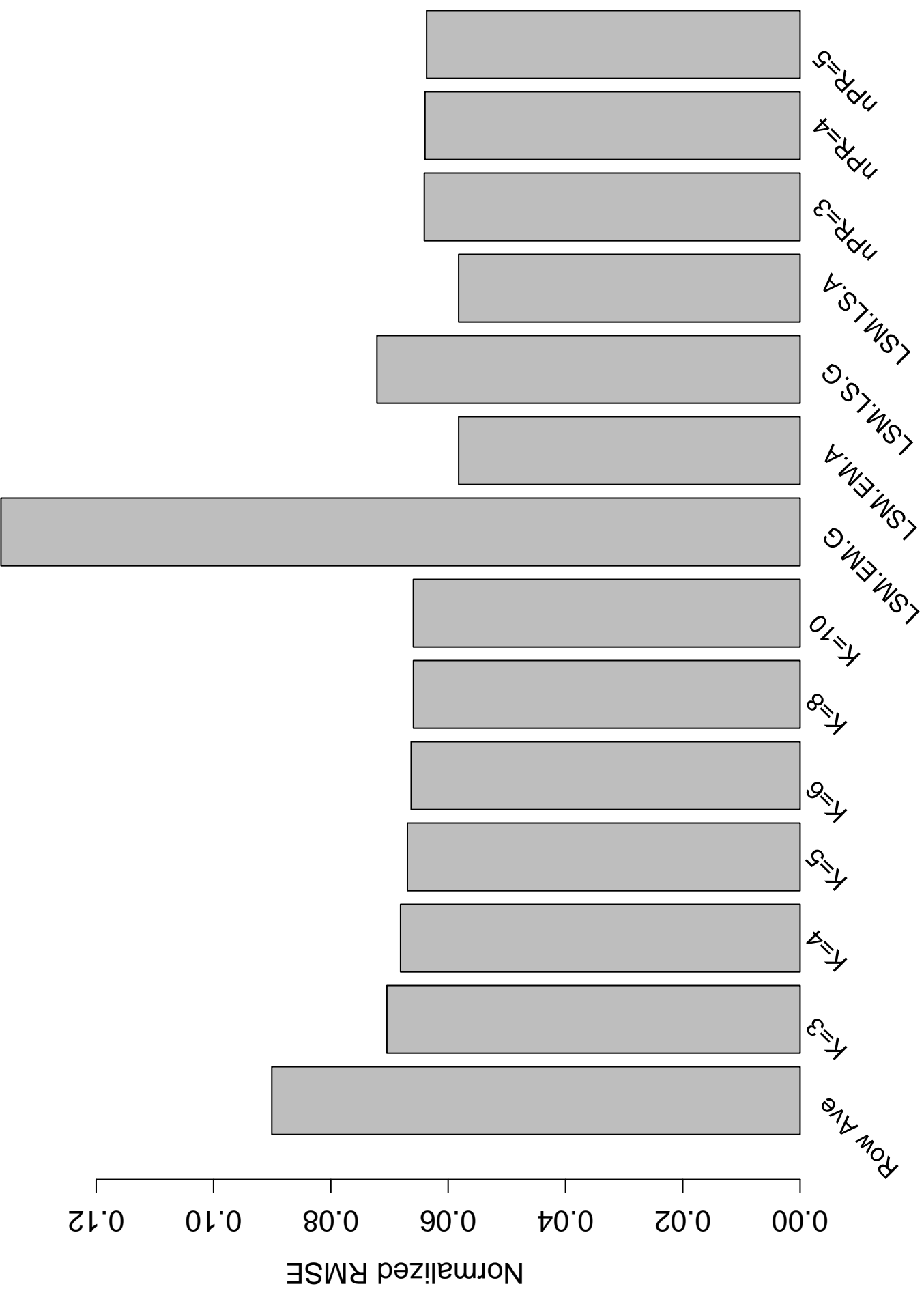

Supplement: Additional file 3 — Figure S3. Coling Dataset: Comparison of k nearest neighbor (KNN), Row Average, Least Squares Methods (LSM), and NIPALS imputation methods on the dataset in [22]. 500 simulations were performed, where each simulation generated a dataset containing 20% missing values by randomly removing spot values from the complete matrix of 343 protein spots. Missing values were imputed using row average (Row Ave), the KNN method with different k nearest neighbor values, or LSM method and the results compared using the normalized root mean square error (RMSE). One set of LSM options allow the user to choose a correlation between protein spots estimated via least squares (LSM.LS.G) or via the EM algorithm (LSM.EM.G). Another set of LSM options allows the user to choose a correlation between arrays estimated via least squares (LSM.LS.A) or via the EM algorithm (LSM.EM.A). Lastly, the user is allowed a combined (array and spot) correlation (LSM.LS.C) and adaptive (LSM.LS.Ad) correlation procedure. The NIPALS methods are summarized by "nPR" which denotes the number of principal components used to impute the missing data. [file 1477-5956-8-66-S3.PDF]

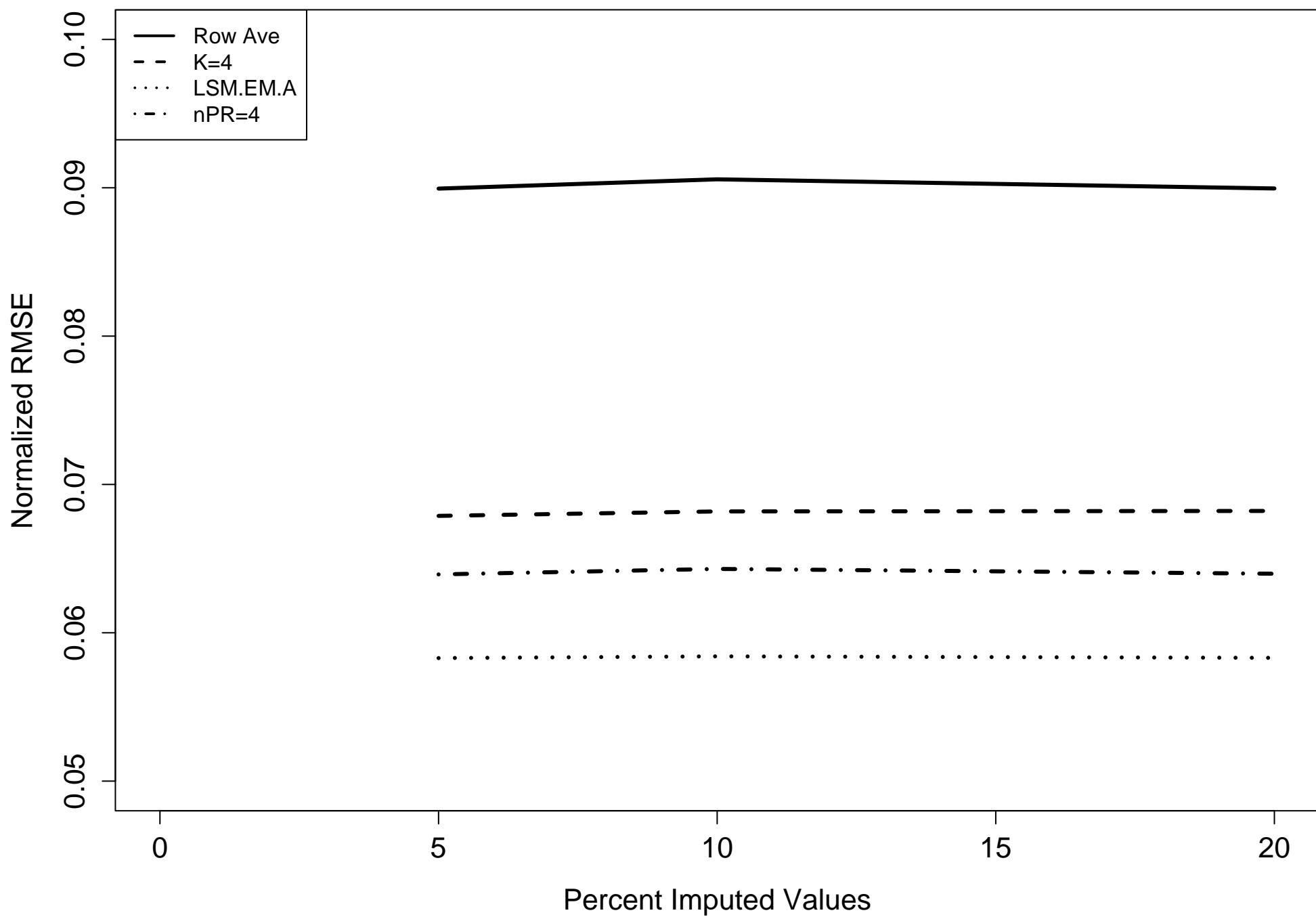

Supplement: Additional file 4 — Figure S4. Coling Dataset: Effects of the amounts of missing data on imputation using Row Average, Least Squares Methods (LSM), KNN and NIPALS imputations methods in the dataset in [22]. 500 simulations were performed, where each simulation generated a datasets containing 5%, 10%, and 20% missing values by randomly removing spot values from the complete data set of 343 protein spots. Missing values were imputed by row average (Row Ave), LSM, and KNN methods with k = 4. The NIPALS methods are summarized by "nPR" which denotes the number of principal components used to impute the missing data. Results of the imputation were compared using RMSE. [file 1477-5956-8-66-S4.PDF]

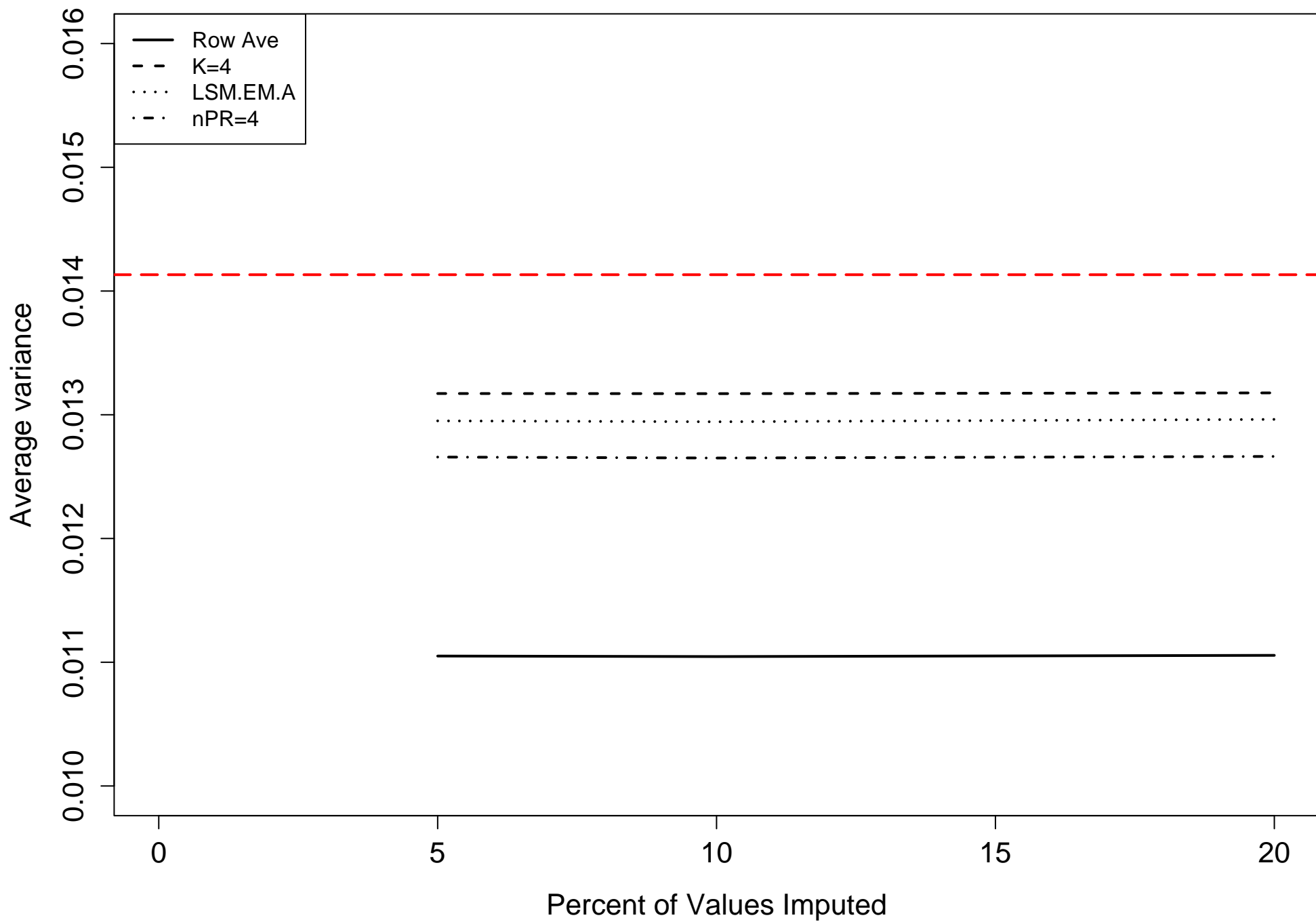

Supplement: Additional file 5 — Figure S5. Coling Dataset: Effects of imputation on the average variance for the dataset in [22]. 500 simulations were performed, where each simulation generated a dataset that contained 5%, 10%, and 20% missing-ness by randomly removing spot values from the complete dataset. Missing values were imputed by row average (Row Ave), LSM, or k nearest neighbors (KNN) imputation with k = 4. The NIPALS methods uses four principal components to impute the missing data. Average variances of the complete 343 protein spots without missing data (red horizontal dotted-dashed line) and after imputation are shown. [file 1477-5956-8-66-S5.PDF]

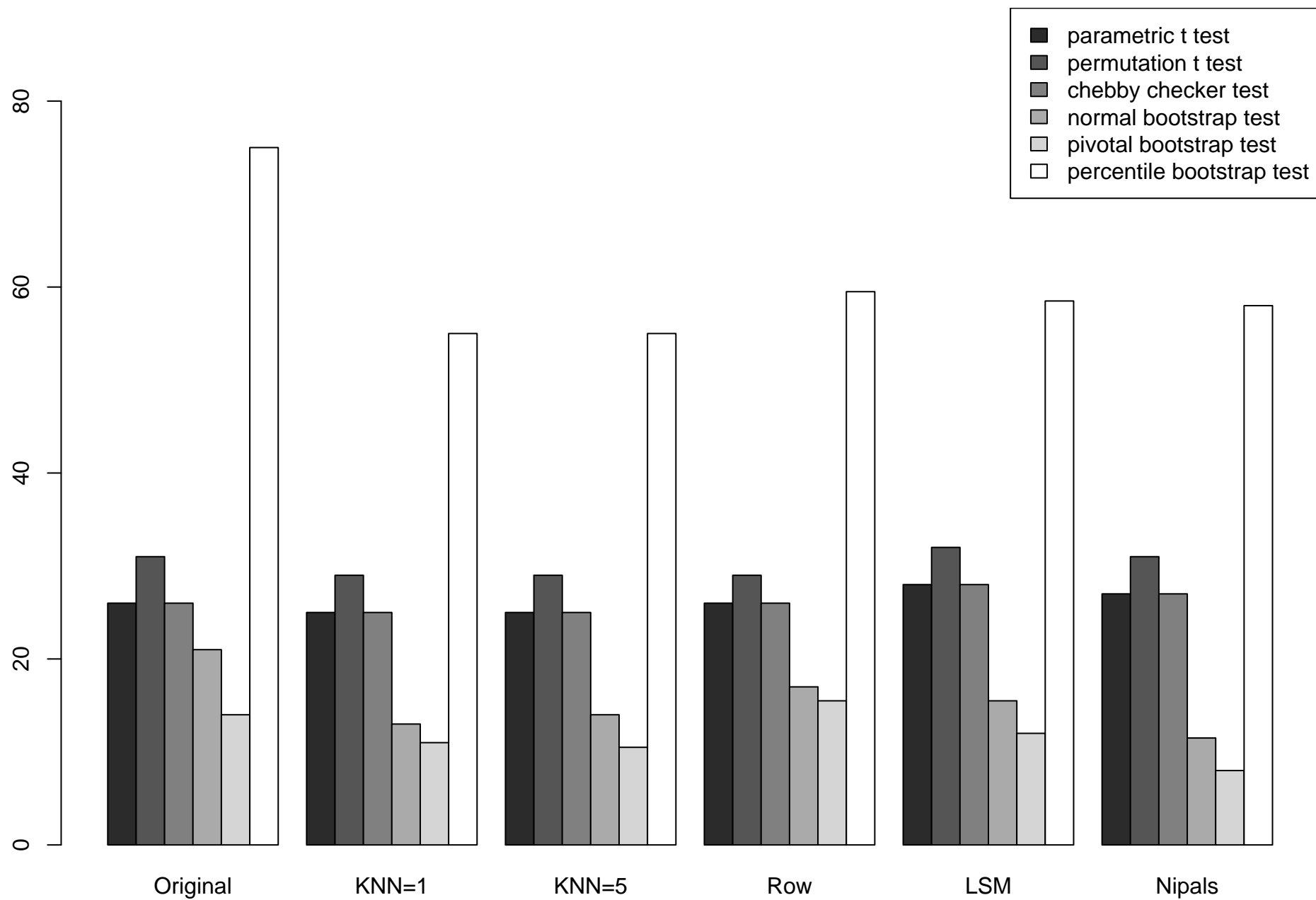

Supplement: Additional file 6 — Figure S6. Coling Dataset: Median number of spots discovered using each method compared against the number of discoveries on the complete dataset in [22]. Randomly 10% of the data was removed and imputed using each method and test for significance was a p-value < 0.05. For the LSM method, we used the LSM option "EMimpute_array". For the NIPALS methods four principal components were used to impute the missing data. [file 1477-5956-8-66-S6.PDF]

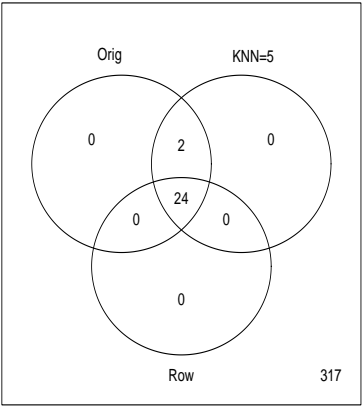

(a)

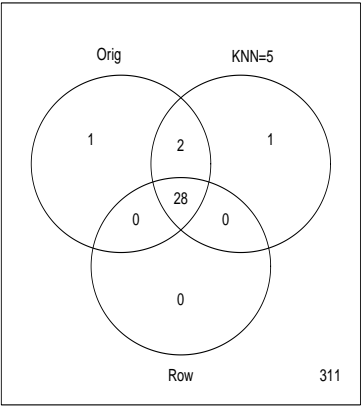

(b)

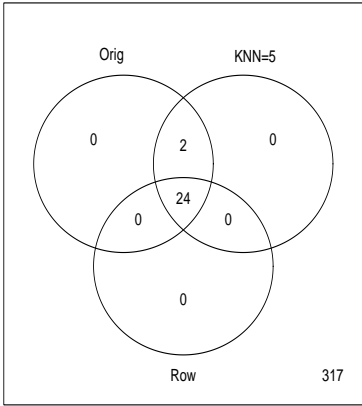

(c)

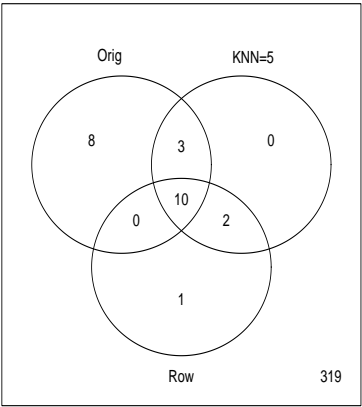

(d)

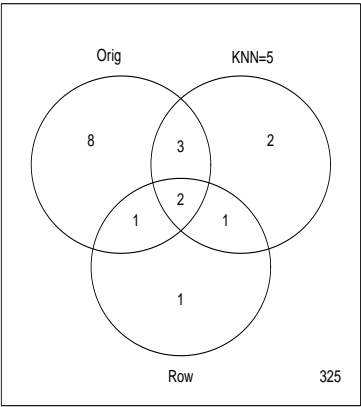

(e)

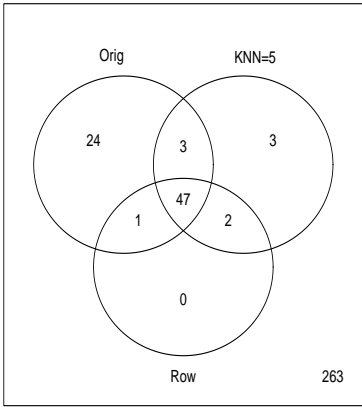

(f)

Supplement: Additional file 7 — Figure S7. Coling Dataset: Summary of Significant Spots on Coling Dataset: Multiple (20) datasets containing 10% missing values were generated by randomly removing spots values from the dataset containing 343 proteins. Missing values were then imputed using the RA, LSM ("EMimpute_array"), or KNN method with k = 5. Values in the Venn diagrams represent the number of discovered proteins (p-value < 0.05) in the original complete dataset and the imputed datasets. Note, to be discovered in KNN method or Row Average method for the imputed datasets, the spot needed to have a p-value less than 0.05 in at least half of the simulated datasets. The Venn diagrams refer to (a) parametric t test, (b) permutation t test, (c) Chebby Checker test, (d) normal-based bootstrap t test, (e) pivotal-based bootstrap t test, (f) percentile-based bootstrap t test. [file 1477-5956-8-66-S7.PDF]

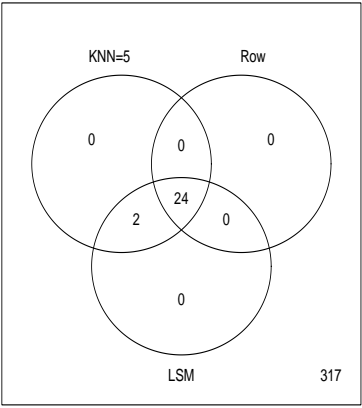

(a)

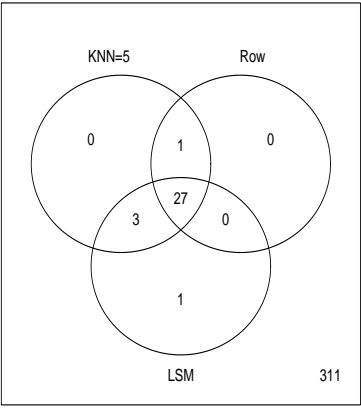

(b)

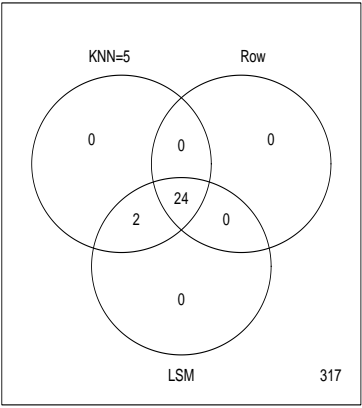

(c)

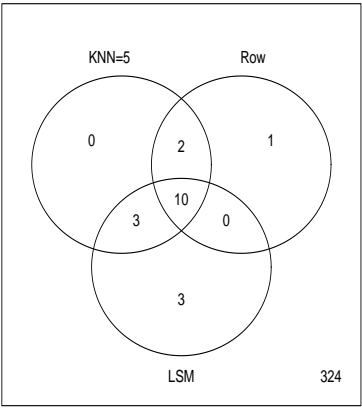

(d)

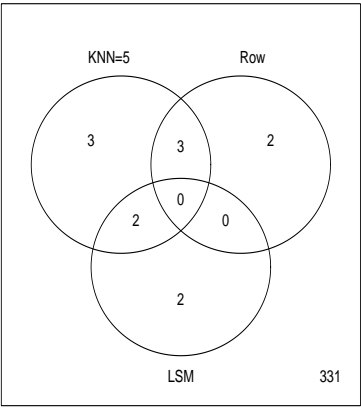

(e)

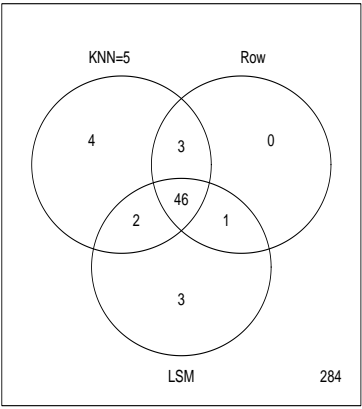

(f)

Supplement: Additional file 8 — Figure S8. Coling Dataset: Summary of Significant Spots on Coling Dataset: Multiple (20) datasets containing 10% missing values were generated by randomly removing spots values from the dataset containing 343 proteins. Missing values were then imputed using the Row Average method, LSM ("EMimpute_array"), or KNN method with k = 5. Values in the Venn diagrams represent the number of discovered proteins (p-value < 0.05) in the original complete dataset and the imputed datasets. Note, to be discovered in KNN, RA, or LSM method for the imputed datasets, the spot needed to have a p-value less than 0.05 in at least half of the simulated datasets. The Venn diagrams refer to (a) parametric t test, (b) permutation t test, (c) Chebby Checker test, (d) normal-based bootstrap t test, (e) pivotal-based bootstrap t test, (f) percentile-based bootstrap t test. [file 1477-5956-8-66-S8.PDF]
